# New paradigm old thinking: the case for emergency obstetric care in the prevention of maternal mortality in Nigeria

**DOI:** 10.1186/1472-6874-10-6

**Published:** 2010-02-17

**Authors:** Kayode T Ijadunola, Macellina Y Ijadunola, Olapeju A Esimai, Titilayo C Abiona

**Affiliations:** 1 Department of Community Health, College of Health Sciences, Obafemi Awolowo University, Ile-Ife, Nigeria; 2 Department of Community Health, Obafemi Awolowo University Teaching Hospitals Complex, Ile-Ife, Nigeria

## Abstract

**Background:**

The continuing burden of maternal mortality, especially in developing countries has prompted a shift in paradigm from the traditional risk assessment approach to the provision of access to emergency obstetric care services for all women who are pregnant. This study assessed the knowledge of maternity unit operatives at the primary and secondary levels of care about the concept of emergency obstetric care (EmOC) and investigated the contents of antenatal care (ANC) counseling services they delivered to clients. It also described the operatives' preferred strategies and practices for promoting safe motherhood and averting maternal mortality in South-west Nigeria.

**Methods:**

The study population included all the 152 health workers (doctors, midwives, nurses and community health extension workers) employed in the maternity units of all the public health facilities (n = 22) offering maternity care in five cities of 2 states. Data were collected with the aid of a self-administered, semi-structured questionnaire and non-participant observation checklist. Results were presented using descriptive statistics.

**Results:**

Ninety one percent of the maternity unit staff had poor knowledge concerning the concept of EmOC, with no difference in knowledge of respondents across age groups. While consistently more than 60% of staff reported the inclusion of specific client-centered messages such as birth preparedness and warning/danger signs of pregnancy and delivery in the (ANC) delivered to clients, structured observations revealed that less than a quarter of staff actually did this. Furthermore, only 40% of staff reported counseling clients on complication readiness, but structured observations revealed that no staff did. Only 9% of staff had ever been trained in lifesaving skills (LSS). Concerning strategies for averting maternal deaths, 70% of respondents still preferred the strengthening of routine ANC services in the health facilities to the provision of access to EmOC services for all pregnant women who need it.

**Conclusion:**

We concluded that maternity unit operatives at the primary and secondary care levels in South-west Nigeria were poorly knowledgeable about the concept of emergency obstetric care services and they still prioritized the strengthening of routine antenatal care services based on the risk approach over other interventions for promoting safe motherhood despite a global current shift in paradigm. There is an urgent need to reorientate/retrain the staff in line with global best practices.

## Background

An estimated 500,000 or more women still die each year from complications of pregnancy and childbirth [1]. Some 99% of these deaths occur in the developing countries, where a woman's lifetime risk of dying from pregnancy and related complications is almost 40 times greater than that of her counterparts in developed countries [2]. By all accounts, progress in reducing maternal mortality has been very slow in developing countries. The vast majority of maternal deaths are due to direct obstetrical complications, including haemorrhage, infection, eclampsia, obstructed labour and unsafe abortion. Twenty years ago, a global Safe Motherhood Initiative was launched to reduce the burden of maternal mortality, especially in developing countries. The initiative at inception, unfortunately, took a few strategic missteps [3]. Emphasis was unduly placed on antenatal care (ANC), including the screening for risk factors, and the training of traditional birth attendants (TBAs) to use safe, hygienic practices. It has since been found out that neither approach had any significant effects on the burden of maternal mortality, as majority of obstetric complications tend to occur in women categorized as low risk, and TBAs can by themselves do very little to save women's lives during serious obstetric complications [3].

Today, strategies are more appropriately focused. It is essential that pregnant women in whom complications develop have access to the medical interventions of emergency obstetric care (EmOC) to ensure favourable maternal and foetal outcomes. Programmes to make such care more widely available include upgrading the infrastructure of community health centers and referral hospitals, and providing necessary and essential drugs, supplies and equipment for the timely delivery of services at all hours. In addition, staff with the appropriate obstetric training and skills in sufficient numbers should be deployed to facilities that offer maternity services. The referral systems between communities and health facilities also need to be strengthened.

The Nigerian National Reproductive Health Policy of 2001 [4] targeted a 50% reduction in maternal mortality from a national average of about 800 deaths to 400 deaths/100,000 live births between 2001 and 2006, a 50% increase in access to safe blood transfusion services, EmOC for women of reproductive age and reproductive health information and services. Nigeria is also a signatory to the Millennium Development Goals (MGDs) of the United Nations member countries, goal 5 of which targets the reduction of maternal mortality by 75% between 1990 and 2015 [5], and the African Roadmap for the accelerated achievement of this goal [6]. The country is however not on track towards the realization of this goal. Recent estimates of the maternal mortality burden by the Federal Ministry of Health [2] puts the maternal mortality ratio at an unacceptably high figure of 800/100,000 live births, even by a developing African country's standard. While innovative programmes are ongoing in a number of states and local government areas with the support of UN agencies, bilateral donors, Non-Governmental Organizations and the private sector, there is no concomitant political will on the part of political leaders who control state and local resources, especially with regards to the placement, training and retention of skilled staff in health facilities [7]. It is therefore difficult to translate globally proven and effective remedies and technologies into action.

The knowledge base of staff in the health facilities and those of the policy makers at the state and local government levels with regard to the paradigm shift, from the Antenatal Risk Assessment approach and promotion of TBA-assisted delivery to the provision of access to EmOC services and delivery under 'skilled birth attendants' for all pregnant women is questionable. However, no previous studies were found in the literature in this direction beyond anecdotal information. To this end, this study was conducted to examine the awareness of maternity unit operatives in public hospitals in five cities of Southwest Nigeria about the change in paradigm and also assess how much of the paradigm shift they adopt in their obstetric pratice. It assessed the maternity unit operatives' knowledge of the concept of EmOC, examined the contents of ANC counseling services they delivered to clients and described operatives' preferred strategies and practices for promoting safe motherhood and preventing maternal mortality among obstetric clients.

## Methods

### Study location

The study was carried out in Osun and Ekiti States of Southwest Nigeria. The study site in Osun State was Ile-Ife (incorporating Ife Central and Ife East Local Government Areas) while four towns in Ekiti State (Ado, Ijero, Ikole and Ikere Ekiti) were selected for study. Data were collected from all the primary and secondary public health facilities that offer obstetric care services. In all, a total of 22 facilities were included, comprising five secondary care (general) hospitals and 17 health centers (both primary and comprehensive).

### Study design

The study employed a descriptive design, exploring the perceptions, knowledge and practices of the operatives of maternity units about various issues pertaining to Safe Motherhood. There was no sampling as all public primary and secondary health facilities in the study location were included.

### Study population

This consisted of all 152 health workers (doctors, midwives, nurses and community health extension workers (CHEWs)) employed in the maternity units of all the health facilities. The antenatal clinics and labour wards of the primary health centers are largely manned by the CHEWs (and occasionally by nurse/midwives), while those of the general hospitals are manned by nurse/midwives backed by doctors. Whichever officer is available at each service delivery point delivers the spectrum of care services required by clients including prenatal care, delivery and EmOC.

### Data collection techniques

Data collection employed both quantitative and qualitative techniques over a four-week period. The quantitative method consisted of a self-administered, semi-structured questionnaire that was applied to the staff while at work. The questionnaire (Additional file 1) was divided into 2 sections - one on the bio-data of the respondents and the other on their knowledge, perceptions and practices regarding EmOC and Safe Motherhood. Knowledge questions required respondents to list the components of Safe Motherhood and Emergency Obstetric Care signal functions, comment on the effectiveness of ANC in predicting and preventing pregnancy-related complications and death, and list the important elements of client-centered ANC for effective maternal mortality reduction. Furthermore, respondents were asked of their awareness of the Lifesaving Skills (LSS) training scheme and were required to mention some of the competencies that the LSS training may help impart. They were also asked if they had been trained in LSS, where trained, when trained and when last they attended any refresher training for upgrading their safe motherhood skills.

The qualitative method consisted of non-participant observation of maternity staff during ANC clinic sessions using a structured checklist. The first two authors (both are doctors while the first author is also a health sociologist) carried out the structured observations. In all, ten ANC clinic sessions (in the 5 general hospitals and 5 health centers) were observed. Of interest during observations were the contents of the ANC messages delivered to clients, staff attitudes and disposition and interpersonal communication between staff and clients.

### Data analysis

Data were analyzed using the Statistical Package for Social Sciences (SPSS) software, version 11. Descriptive statistics were used to summarize the data and were presented using frequency tables and percentages. To assess the respondents' knowledge of EmOC, relevant questions from the questionnaire had weights attached to them to create a composite score of knowledge. Maximum score obtainable was 14 points and points were awarded on a discrete (whole number) rather than a continuous scale, based on the number of positive responses.

Interpretation of scores was based on the Nigerian university education scoring system. Respondents whose scores translated into 70% or more were classified as having excellent knowledge of EmOC; those who scored between 50% and 69% were classified to have good knowledge; those who scored between 40% and 49% were classified to have fair knowledge while those whose scores were below 40% were classified as having poor knowledge. For example, if one scored 5 out of 14, (36%), he/she was interpreted to have poor knowledge, 9 out of 14 (64%), was interpreted to have good knowledge while if he/she scored 10 out of 14 (71%), the knowledge was interpreted as excellent. For knowledge of LSS competencies, every correct LSS skill/competency volunteered attracted a point (to a maximum of 6 points). Any respondent who volunteered 3 points (50%) was reported to have good knowledge while those who volunteered 2 (33%) or less points were reported to have poor knowledge.

## Results

### Sample demographics

One hundred and fifty two respondents consisting of 21 doctors (13.8%), 57 nurses/midwives (37.5%) and 74 CHEWs (48.7%) participated in the study. One hundred and seventeen (77%) of the respondents were females, as a majority of the respondents were either Community Health Extension Workers (CHEWs) or nurses, both of which are female dominated professions in Nigeria, while 35 (23%) were males. The mean age (SD) of the respondents was 36.2 (2.2) years with a modal age range of 30-39 years.

### Knowledge of EmOC

The general knowledge of the respondents concerning the concept of EmOC was poor (Table 1). No respondent had an excellent knowledge of the issues; only one respondent (0.7%), a doctor, had good knowledge, 13 people (8.5%), also mostly doctors, had a fair knowledge, while about 91% had poor knowledge. There was no difference in EmOC knowledge of respondents across age groups (results not shown); besides, the youngest respondent was 28 years old while the oldest was 58 years and so, most were not fresh graduates, but practitioners who had been in practice for some time, entrenched in the old paradigm.

**Table 1:** Maternity unit workers' knowledge of the concept of Emergency Obstetric Care

Level of Knowledge	Doctors (n = 21) Freq (%)	Nurses (n = 57) Freq (%)	*CHEWs (n = 74) Freq (%)	Total (n = 152) Freq (%)
Good	1 (4.8)	0 (0)	0 (0)	1 (0.7)
Fair	9 (42.8)	3 (5.3)	1 (1.4)	13 (8.5)
Poor	11 (52.4)	54 (94.7)	73 (98.6)	138 (90.8)
Total	21 (100)	57 (100)	74 (100)	152 (100)

*CHEWs = Community Health Extension Workers

The contents of the ANC counseling services delivered by the respondents to their clients are presented in table 2. A significant proportion of the respondents reported that they included facts that can make the outcome of pregnancy safer in their ANC talks. About 63% reported that they counsel clients that all pregnancies may be at risk of developing complications; 71% reported counseling clients that the occurrence of maternal complications may not be accurately predicted in individual women. Similarly, about 63% of the respondents reported that they counsel clients on birth preparedness, 75% on warning and danger signs of pregnancy and delivery and 83% on post-partum family planning. However, only 40% of the respondents, more doctors than the other health workers, reported counseling clients on complication readiness. Generally, the doctors and nurse/midwives faired better than the CHEWs on the contents of ANC counseling services. Results of the non-participant observations, however, did not confirm many of these responses as less than a quarter of staff observed counseled clients on the variety of items listed in table 2; furthermore, counseling sessions tended to be more generic than being item-specific.

**Table 2:** Reported contents of antenatal care (ANC) counseling services delivered by maternity unit workers to their clients.

Items reportedly discussed by maternity staff with clients during ANC counseling:	Doctors (n = 21) Freq (%)	Nurses (n = 57) Freq (%)	*CHEWs (n = 74) Freq (%)	Total (n = 152) Freq (%)
That all pregnant women are at risk of maternal complications	16 (76.2)	50 (87.7)	29 (39.2)	95 (62.5)
That Incidence of maternal complications may not be accurately predicted in individual women	13 (61.9)	40 (70.2)	55 (74.3)	108 (71.1)
That maternal complications, though unpredictable are treatable	17 (81.0)	48 (84.2)	50 (67.6)	115 (75.7)
Birth preparedness	13 (61.9)	47 (82.5)	35 (47.3)	95 (62.5)
"Warning/danger" signs of pregnancy and delivery	17 (81.0)	51 (89.5)	46 (62.2)	114 (75.0)
Postpartum family planning	19 (90.5)	50 (87.7)	57 (79.0)	126 (82.9)
Complication readiness	15 (71.4)	20 (35.1)	6 (8.1)	61 (40.1)

*CHEWs = Community Health Extension Workers

### Delivery room practices

The delivery room practices of the respondents are presented in table 3. Only about 41%, more doctors than the other health workers, routinely use the partograph in monitoring the progress of labour. While slightly more than half (52.6%) of the respondents would permit a woman's spouse or partner in the delivery room, only 18% of them would permit one or more relatives apart from the husband, even on the request of the client. Surprisingly, less than half of the respondents reported the use of obstetric protocols to aid decision making in the labour ward, and this was less so for the nurse/midwives and the CHEWs compared with the doctors. This was despite the fact that less than 10% of the respondents had received any training in Life Saving Skills (LSS), table 4.

**Table 3:** Delivery room practices of maternity workers

Delivery room practices engaged in by maternity staff	Doctors (n = 21) Freq (%)	Nurses (n = 57) Freq (%)	*CHEWs (n = 74) Freq (%)	Total (n = 152) Freq (%)
Allow client's husband into the delivery room	12 (57.1)	34 (59.6)	34 (45.9)	80 (52.6)
Allow one or more client's relatives (apart from the husband) into the delivery room	6 (28.6)	13 (22.8)	8 (10.8)	27 (17.8)
Use the partograph in monitoring labour	18 (87.5)	35 (61.4)	9 (12.2)	62 (40.8)
Refer to obstetric protocols in the labour ward to aid decision making	11 (52.4)	27 (47.4)	27 (36.5)	65 (42.8)

*CHEWs = Community Health Extension Workers

**Table 4:** Maternity unit workers' exposure to skills/training toward ensuring "safe motherhood"

Safe Motherhood skills/training that maternity staff are exposed to	Doctors (n = 21) Freq (%)	Nurses (n = 57) Freq (%)	*CHEWs (n = 74) Freq (%)	Total (n = 152) Freq (%)
Awareness of *LSS training	18 (85.7)	22 (38.6)	4 (5.4)	44 (28.9)
Knowledge of competencies that the LSS training confers on trainees				
- Good knowledge	6 (28.6)	1 (1.7)	0 (0)	7 (4.6)
-Poor knowledge	14 (66.7)	54 (94.7)	74 (100)	142 (93.4)
Number of staff trained in LSS	7 (33.3)	6 (10.5)	1 (1.3)	14 (9.2)
Attendance of staff at other trainings for upgrading of skills in safe motherhood				
-Never	16 (76.2)	51 (89.5)	65 (87.8)	132 (86.8)
- In the last 1 year	1 (4.8)	2 (3.5)	3 (4.05)	6 (3.9)
- In the last 2-5 years	4 (19.0)	4 (7.0)	6 (8.11)	14 (9.2)

*CHEWs = Community Health Extension Workers

**LSS = Lifesaving skills

The awareness of LSS training was quite poor among the respondents as only 29% of them were aware of the scheme, and the bulk of these were doctors (Table 4). It was therefore not surprising that a majority (93%) of the respondents had poor knowledge of the competencies acquired through the LSS training. Similarly, a majority of the respondents had never attended any competency based training programme in the area of safe motherhood since their graduation from basic training (Table 4). Most of the respondents, however, displayed great zeal and enthusiasm to get trained and be updated on current issues with regards to safe motherhood and maternal mortality reduction interventions, even if they would have to co-fund such training with their employers (results not shown). The consensus opinion among respondents, however, was that their employers were not interested in funding their attendance at updates and workshops.

### Preferred Strategies for averting maternal deaths

Concerning strategies for averting maternal deaths (Table 5), 70% of respondents would prefer the strengthening of routine ANC services in the health facilities to the provision of access to EmOC services for all pregnant women who need it. This view was stronger among the nurse/midwives and CHEWs compared with the doctors. The EmOC strategy was preferred by only 21% of respondents while just 6% of them reported the improvement in referral and other support services as their preferred strategy for averting maternal deaths. Within the three months preceding the survey, almost 62% of the respondents reported that they had not provided any emergency obstetric care services to clients (Table 6). About 22% reported that they had partaken in the delivery of basic emergency obstetric care services while about 16% reported offering comprehensive emergency obstetric care services to clients during the same period, and a majority of these were doctors and included no CHEWs. About 82% of the respondents however reported providing post-partum family planning services during this period.

**Table 5:** Maternity unit workers' preferred strategies for averting maternal mortality

Reported Preferred Strategies	Doctors (n = 21) Freq (%)	Nurses (n = 57) Freq (%)	*CHEWs (n = 74) Freq (%)	Total (n = 152) Freq (%)
Strengthening routine ANC services	10 (47.6)	43 (75.4)	54 (73)	107 (70.4)
Improved referral and other support services	1 (4.8)	0 (0)	8 (10.8)	9 (5.9)
Access to **EmOC for all pregnant women	9 (42.9)	13 (22.8)	10 (13.5)	32 (21.1)

*CHEWs = Community Health Extension Workers

**EmOC = Emergency Obstetric Care

**Table 6:** Maternity unit workers' provision of "Safe Motherhood" services in the 3 months preceding the study

Types of Safe Motherhood services offered	Doctors (n = 21) Freq (%)	Nurses (n = 57) Freq (%)	*CHEWs (n = 74) Freq (%)	Total (n = 152) Freq (%)
No *EmoC	3 (14.3)	32 (56.2)	59 (79.7)	94 (61.8)
Basic EmOC	2 (9.5)	17 (29.8)	15 (20.3)	34 (22.4)
Comprehensive EmOC	16 (76.2)	8 (14.0)	0 (0)	24 (15.8)
Post-abortal Care	18 (85.7)	31 (54.4)	1 (1.4)	50 (32.9)
Postpartum Family Planning	20 (95.2)	42 (73.7)	62 (83.8)	124 (81.6)

*CHEWs = Community Health Extension Workers

**EmOC = Emergency Obstetric Care

## Discussion

The continuing burden of maternal mortality, especially in developing countries has prompted a shift in paradigm from the traditional risk assessment approach of attempting to predict and prevent maternal deaths and complications to the provision of access to emergency obstetric care services for all women who are pregnant, (and might thus experience complications) and the treatment of such complications [8]. The basic requirements for provision of emergency obstetric functions have been delineated and the essential elements of obstetric care at the first referral level have been documented [9]. New training needs for maternity care providers at hospitals and peripheral level facilities have also been recognized, and a number of initiatives attempting to meet those needs [10], especially in resource-constrained settings, have been similarly proposed. While the audience for training varies from project to project, "skilled birth attendants" as defined by the World Health Organization is often identified as the appropriate focus for training [11]. We now know enough about what works to implement preventive community-based strategies to improve maternal and newborn health by 2015.

In resource-poor settings, life-threatening obstetric complications still often go unidentified and pregnancy complications are frequently not properly managed by health services. The requirements, considered to be the minimum in the provision of care at the district or sub-district hospitals or health centers, are also seriously deficient in many countries, Nigeria inclusive, where these qualities sometimes do not even exist at the teaching hospital level [8]. In this study, maternity unit operatives lacked the necessary post-basic training to equip them for the effective delivery of safe motherhood services in the face of the current paradigm shift. Schemes such as the LSS training were strange to many of them and their knowledge of what constitutes emergency obstetric care services, LSS competencies and major safe motherhood interventions was therefore unsurprisingly poor. These findings were however not far fetched. Although, the shift in paradigm already forms part of the current basic training of doctors and midwives in Nigeria, it is only true of the recent times. The practitioners surveyed in this study had left school for some time and were not so trained in the change of paradigm. Their only possible source of information is retraining on the job and this too has not been vigorously pursued by employers as confirmed by study participants.

Even though the respondents reported incorporating the contents of client-centered ANC services into their ANC counseling sessions, non-participant observations by the investigators did not confirm this. The staff probably wanted to identify with global best practices, as depicted in the research instrument, but were being constrained by a number of factors that may include technical incompetence as well as policy and administrative bottlenecks. This finding further gave credence to the use of structured observations in this study as against the sole use of the questionnaire survey which might have introduced a social desirability bias about the reported practices of the respondents.

While the respondents were enthusiastic about post-basic training and were even willing to co-fund their attendance at such trainings, they were often constrained by the disposition of their employers who were reported not to be passionate about funding the attendance of staff at updates and workshops. The employers (officials of the health departments of the states and LGAs) routinely reiterate the unacceptably high maternal mortality burden of the country and the official commitment of the state to several of the international policy documents targeting the reversal of the trend, including the millennium declaration of the United Nations. They however fail to make improved maternal health a political priority by deploying the necessary resources to meet targets [7]. The excuse often advanced is that it is expensive to employ skilled staff to man primary health facilities (in the face of scarce state/LGA resources) [7]. This was also possibly why a majority of the respondents (CHEWs) in this study who manned maternity units in primary level public health facilities were "unskilled birth attendants" by international standards. This category of health workers, by design, have little or no basic training in obstetric care and are supposed to spend part of their working day in the community and part in the health facilities attending to minor ailments and injuries [12]. They have however become the "de facto" health workers in primary health facilities in Nigeria, including maternity units. This situation has grave implications for the fight against maternal mortality in the country as the workers in whose care the health of women and mothers are committed are not "skilled".

The delivery room practices of the respondents were to a large extent a reflection of their level of training (both basic and post-basic). A majority of them will not allow the relatives of pregnant women into the labour ward to support them in labour. Similarly, operatives (especially the non-physicians) neither used the partograph to monitor labour nor used obstetric protocols to aid decision making. This could be as a result of poor monitoring and supervision of the staff, lack of skills in the use of partographs and protocols or even shortage in the supply of these tools. The situation however has implications for utilization of obstetric services by women and their families as the tendency of the clients is to prefer services that respect their wishes to have relatives in attendance at deliveries and those that give them some attention and a semblance of intensive care. Regrettably for the formal health system in Nigeria, the TBAs and the faith-based providers of care incorporate such "humane" principles into their package of care, and are therefore more endeared to the women and their families, despite their limited technical skills and competences.

It was further observed that the preferred strategy for improved maternal outcomes by a majority of the maternity unit operatives in this study was the strengthening of routine ANC services ahead of "access to EmOC services by all women that need them" and "improved referral services". Besides, EmOC functions were not routinely performed by the health workers. It could therefore be inferred that these operatives still believed in the teaching of "if we take care of pregnant women very well during ANC and identify those at risk, they will be well". While ANC is very useful and should continue to be promoted as a strategy for improved health facility utilization during pregnancy and delivery, and improved maternal and fetal outcomes, it must be "focused/client centered" and deemphasize individual risk assessment. Current best practices suggest that we categorise every pregnant woman as being at risk of possible obstetric complications and death [8,13,14] and should therefore be given access to EmOC services based on need. These operatives will need a total reorientation/retraining if they must embrace the paradigm shift and adopt best global practices for improved maternal outcomes.

This study was not without some limitations. While the authors upheld every ethical standard to ensure accurate reporting of practices by the health workers, and even conducted some non-participant observations, we still acknowledge the possibility of over-reporting of perceived best practices while under-reporting practices perceived to be unacceptable by the health workers. The inability to observe deliveries and possibly EmOC sessions while the study lasted is most regretted. However, ANC was the most routine of all the safe motherhood services being provided by the health facilities, and it was the one with the most predictable time schedule for observation. Being present at deliveries or other service sessions would have been most accidental, depending on the time of visit to the health facility for observations. While this study was conducted in two states of southwest Nigeria, the results might be generalizable only to the rest of the southwest region that share similar and comparable health indices. Similar studies in the other five regions of the country, where health indices are worse than in the southwest, are likely to generate worse outcomes, and are worth conceiving.

## Conclusion

We concluded that maternity unit operatives at the level of primary and secondary care in south-west Nigeria were poorly knowledgeable about the concept of emergency obstetric care services and still preferred the strengthening of routine antenatal care services based on the risk approach for promoting safe motherhood and reducing maternal mortality, despite a current shift in paradigm that is globally acceptable. There is an urgent need to make improved maternal health a state priority in Nigeria so that health workers of the right number, mix and training/orientation can be employed and deployed to man maternity units at the various levels of care. Advocacy efforts of women's groups, community organizations and the mass media are urgently needed in this direction.

## Competing interests

The authors declare that they have no competing interests.

## Authors' contributions

KTI conceived of the study, participated in its design, data collection, coordination and analyses and co-wrote/edited the draft manuscript. MYI participated in the study design, data collection, writing and editing of the manuscript. OAE participated in the study design, data collection, manuscript writing and editing. TCA participated in data collection, analysis, manuscript writing and editing. All authors read and approved the final manuscript.

## Authors' information

Kayode T Ijadunola, MBChB, MSc, FWACP - Associate Professor, Department of Community Health, Obafemi Awolowo University, Ile-Ife, Nigeria

Macellina Y Ijadunola, MBChB, MPH - Senior Registrar, Department of Community Health, Obafemi Awolowo University Teaching Hospitals, Ile-Ife,Nigeria

Olapeju A Esimai, MBChB, MSc, FMCPH - Senior Lecturer, Department of Community Health, Obafemi Awolowo University, Ile-Ife, Nigeria

Titilayo C Abiona, MBChB, FMCPH - Senior Lecturer, Department of CommunityHealth, Obafemi Awolowo University, Ile-Ife, Nigeria

## Pre-publication history

The pre-publication history for this paper can be accessed here:

http://www.biomedcentral.com/1472-6874/10/6/prepub

## Supplementary Material

Additional file 1 **Study Questionnaire**. New paradigm old thinking: the case for emergency obstetric care in the prevention of maternal mortality in Nigeria Click here for file
