# Supplementary material for: New paradigm old thinking: the case for emergency obstetric care in the prevention of maternal mortality in Nigeria
Source: BMC Womens Health. 2010 Feb 17;10:6. doi: 10.1186/1472-6874-10-6 (PMC2836278; doi:10.1186/1472-6874-10-6)
Supplement: Additional file 1 — Study Questionnaire. New paradigm old thinking: the case for emergency obstetric care in the prevention of maternal mortality in Nigeria [file 1472-6874-10-6-S1.DOC]

**New Paradigm, Old Thinking: The Case for Emergency Obstetric care in the Prevention of Maternal Mortality in Nigeria**

**Study Questionnaire**

**Section A: Biodata**

1. Age last birthday ……………..(yrs.)
2. Sex: Male [ ]

Female [ ]

1. Marital Status: Single [ ]

Married [ ]

Divorced [ ]

Separated [ ]

Widowed [ ]

1. Profession (please specify)……………………………….
2. Designation……………………………………………….
3. Number of years since basic qualification……………… (yrs.)
4. Number of years in the practice of present specialty…………(yrs.)

**Section B: Perceptions and Practices**

1. Have you heard of the term “Safe Motherhood? Yes [ ]

No [ ]

1. If yes, list the major components of safe motherhood

……………………………………………………

…………………………………………………….

……………………………………………………

……………………………………………………

……………………………………………………

1. How effective is prenatal (antenatal) care in predicting pregnancy related complications and death?

i. Not effective [ ]

ii. Barely effective [ ]

iii. Fairly effective [ ]

iv. Very effective [ ]

1. How effective is prenatal care in preventing pregnancy-related complications and death?

i. Not effective [ ]

ii. Barely effective [ ]

iii. Fairly effective [ ]

iv. Very effective [ ]

1. Which two elements of prenatal care offered to pregnant women without symptoms can effectively reduce the risk of maternal deaths (rank the most important 1 and the next important 2)

i. hygiene education [ ]

ii. nutrition education [ ]

iii. tetanus toxoid immunization [ ]

iv. birth preparedness [ ]

v. antenatal risk screening [ ]

vi. complication readiness [ ]

1. Does the content of the prenatal care that you provide to pregnant women and their families include any of the following?

i. That all pregnant women are at risk of developing pregnancy related

complications? Yes [ ] No [ ]

ii.That health workers may not accurately predict which women will develop life threatening pregnancy-related complications? Yes [ ] No [ ]

iii. That maternal complications though, mostly unpredictable are treatable and need not result in maternal deaths? Yes [ ] No [ ]

iv. Discussions on plans for delivery (e.g. intention of where to deliver, who the pregnant woman wants to be present at her delivery, contingency plans in the event of complications? Yes [ ] No [ ]

v. Signs and symptoms of complications during pregnancy and delivery?

Yes [ ] No [ ]

vi. Post-partum family planning? Yes [ ] No[ ]

1. Have you heard of the term “Emergency Obstetric Care”? Yes [ ] No [ ]
2. If yes, list the components/signal functions of Emergency Obstetric Care that you know

(i)…………………………………………………

(ii)………………………………………………..

(iii)……………………………………………….

(iv)……………………………………………….

(v)………………………………………………..

(vi)……………………………………………….

(vii)……………………………………………….

(viii)………………………………………………

1. Would you allow any of these relatives of a pregnant woman in the delivery room on the client’s request?

(a) Husband Yes [ ] No [ ]

(b) Parents Yes [ ] No [ ]

(c) In-laws Yes [ ] No [ ]

(d) Friends Yes [ ] No [ ]

(e) Siblings Yes [ ] No [ ]

1. Do you routinely use the partograph in monitoring the progress of labour?

Yes [ ] No [ ]

1. If no, why not?
   - 1. Partograph is not available [ ]
     2. Not trained in the use of the partograph [ ]
     3. Use of the partograph is complicated [ ]
     4. Use of the partograph is not necessary [ ]
     5. Others (please specify)_____________________
2. Are there obstetric protocols or guidelines in your facility to guide your decisions during obstetric emergencies? Yes [ ] No [ ]
3. If yes, list some of the one you have

(i)……………………………………………..

(ii)…………………………………………….

(iii)……………………………………………

(iv)……………………………………………

1. Have you heard of the lifesaving skills (LSS) training? Yes [ ] No [ ]
2. If yes, list some of the component topics/skills learnt during the LSS training

(i)……………………………………………..

(ii)…………………………………………….

(iii)……………………………………………

(iv)……………………………………………

(v)…………………………………………….

(vi)…………………………………………….

1. Have you been trained in LSS? Yes [ ] No [ ]
2. If yes, when and where? When……………………Where………………………
3. When last did you attend any other refresher training for upgrading of skills in safe motherhood, apart from the LSS and where was the training?
4. when………………………………………

(ii) where……………………………………..

1. If you have not attended any, why not?_________________________________

_________________________________________________________________

1. Which of the following is your preferred strategy for averting maternal mortality and promoting safe motherhood? Rank the one you consider most effective as 1 and the least effective as 3.

(a) Strengthening prenatal care and antenatal risk assessment [ ]

(b) Development and strengthening of referral and support systems [ ]

© Provision of access to emergency obstetric care for all pregnant women in case they develop complications [ ]

1. Which of the following should be the priority of health planners for averting maternal mortality and promoting safe mothethood? Rank the one you consider most effective as 1 and the least effective as 3.

(a) Strengthening prenatal care and antenatal risk assessment [ ]

(b) Development and strengthening of referral and support systems [ ]

© Provision of access to emergency obstetric care for all pregnant women in case they develop complications [ ]

1. Did you perform any of the following services in your facility in the last three months?

(a) Administration of intravenous antibiotics? Yes [ ] No [ ]

(b) Administration of intravenous oxytocics? Yes [ ] No [ ]

© Administration of intravenous anticonvulsants Yes [ ] No [ ]

(d) Manual removal of placenta? Yes [ ] No [ ]

(d) Removal of retained products of conception? Yes [ ] No [ ]

(e) Assisted vaginal delivery? Yes [ ] No [ ]

(f) Blood transfusion? Yes [ ] No [ ]

(g) Caesarean section? Yes [ ] No [ ]

(h) Post-abortal care Yes [ ] No [ ]

(i) Postpartum family planning Yes [ ] No [ ]
